# Hernia of the Whole Bowel

**Published:** 1875-02-01

**Authors:** H. H. Briggs

**Affiliations:** Chattanooga, Tenn.


### HERNIA OF THE WHOLE BOWEL.

By H. H. Briggs, M.D., of Chattanooga, Tenn.

L. A. R., aged 21 years; native of Tennessee. Farmer's daughter—always healthy and strong; physically, well developed; used to sturdy, out-of-door life and labor, was taken in travail, on January 11th in the evening. After an easy, though somewhat lingering labor, she was delivered, by a midwife, of a child, the unnatural appearance of which, caused the attendants to send for their physician, Dr. J. B. Norris. The child lived thirty hours.

On the morning of the 15th, at Dr. Norris' request, Dr. G. A. Baxter and I went down with him and made an autopsy.

*Post-mortem*, thirty hours after death. Cadaveric rigidity not marked. Body weighed three and one-half pounds, and was apparently that of a seven months' child. No marks or spots of any kind upon it. Dr. Norris assured us that it had not changed perceptibly in appearance since death.

Projecting from the abdomen, there was a tumor as large as two folded fists, bright red in color, and apparently made up of intestine. Tracing the tumor by its pedicle, we found that the latter entered the abdomen, at a quarter of an inch external to the umbilicus, and entirely separate from it.

Examining closely for evidences of a sac, we found none on the body of the tumor, but around its pedicle and extending for one-half inch from the abdominal walls, was attached a clear membrane, which we thought to be the parietal peritoneum, and dissection proved it to be such.

Opening the body, we found the lungs, heart, liver and spleen, natural in size, color and consistence. Following down the alimentary canal, we found the œsophagus and stomach natural, but the duodenum led directly from the pylorus to the opening in the walls of the belly.

Tracing the intestine down, fold by fold, the duodenum, the jejunum, the ileum, and the whole of the large intestine, except the rectum, were found to be extra-mural.

The extruded bowel was thickened, firm, and of a bright, red color. Its serous coat was hardly to be seen or separated from the muscular. We noticed no adventitious membrane, nor any recent lymph.

The muscular coats of the bowel were markedly hypertrophied, showing the condition to have been of long standing. The mesentery was thickened and firm, as is indeed common in old umbilical herniæ. We found eight nodules scattered over it, solid to the touch, yellowish in color, and cheesy on section. The edges of the hernial ring were firm, clearly defined, whitish, and quite cartilaginous under the knife. We could see no granulations or evidences of recent inflammatory action. The seeming inflammatory adhesions between the mural peritoneum and the pedicle of the tumor, together with the glazed, contracted edges of the orifice in the walls of the belly, inclined us to the belief, that the hernia was due to a rupture of the parietes, rather than to a deficiency in them. It was produced at an early period in fœtal life. In none of the few books at our command, do we find mention of a ventral hernia of the whole bowel, occurring in early fœtal life: the child going to term and living for some hours after birth. With our present notions as to the suspension of the child in fluid during interuterine life, it is not easy to see how force could be brought to bear sufficient to produce a traumatic, ventral hernia; yet it seems probable that such was the case in the present instance. We know of no treatment applicable to the case.

The disparity between the development of the abdomen generally, and that of the extruded bowel, would have prevented its return.

It was impossible to close the exploratory incision with the tumor inside, so that enlarging the hernial ring would not have made reduction possible. Taxis would have availed nothing.

